# Postpartum family planning among women attending maternal and child health centers in Assiut Governorate, Upper Egypt

**DOI:** 10.1186/s42506-024-00160-0

**Published:** 2024-06-10

**Authors:** Heba M. Mohammed, Maria A. Zaky, Ahmed M. Hany

**Affiliations:** 1 Public Health and Community Medicine Department, Faculty of Medicine, Assiut University, Assiut, Egypt; 2 Family Planning Physician in Kolta Maternal and Child Health Center, Ministry of Health, Assiut, Egypt

**Keywords:** Child health, Postpartum family planning, Maternal health care, Childbirth

## Abstract

**Background:**

Postpartum family planning (PPFP) is important in helping couples to achieve their reproductive intentions. National surveys have consistently reported higher fertility, lower use of family planning (FP), and higher unmet needs for FP in Upper Egypt. This study aims to identify the factors associated with the use of PPFP in Assiut Governorate, Upper Egypt, and to assess the current status of integration of PPFP counseling in the existing maternal and child health services (MCH).

**Methods:**

The study employed a cross-sectional study design, collecting data from 455 postpartum women aged 15 to 49 years in 4 primary health care (PHC) centers in Assiut Governorate, Upper Egypt. The women were interviewed using a structured questionnaire. The questionnaire consisted of 4 sections: the first section included the participants’ demographic characteristics; the second section included women’s obstetric history; the third section included questions about PPFP knowledge, attitude, past and current use of contraception; and the fourth section assessed the current status of integrating PPFP counseling within antenatal, natal, and postnatal services.

**Results:**

In total, 54.5% of postpartum women were using a modern contraceptive method. The significant predictors of using PPFP methods were as follows: receiving information about PPFP from health care providers (AOR = 11.46, *p* < 0.001), better attitude towards PPFP (AOR = 10.54, *p* < 0.001), using modern FP methods (AOR = 6.98, *p* < 0.001), resumption of menstruation (AOR = 4.11, *p* < 0.001), older age (AOR = 2.15, *p* < 0.05), and better PPFP knowledge (AOR = 1.72, *p* < 0.001). Only 5.3%, 1.3%, and 3.5% received PPFP counseling during antenatal care (ANC), delivery, and the postpartum period, respectively.

**Conclusions:**

Postpartum contraception use was associated with receiving PPFP counseling by health care providers and women’s knowledge and attitude regarding PPFP. However, FP counseling was not integrated with other MCH services. Updating the components of MCH services to include PPFP counseling during ANC, at delivery, and during the postpartum period should be prioritized by program planners and policymakers.

## Introduction

The World Health Organization defines postpartum family planning (PPFP) as the prevention of unplanned and closely spaced pregnancies within the first 12 months after childbirth; 95% of women who are 0 to 12 months postpartum want to avoid pregnancy in the next 24 months but 70% of them are not using contraception [1].

The first year after a woman has given birth is important, as women who are not exclusively breastfeeding (BF) can become pregnant 4 to 6 weeks postpartum, so women who desire to delay a subsequent pregnancy should begin using contraceptive methods immediately after delivery and before the resumption of sexual activity [2, 3]. Breastfeeding women can use many modern family planning (FP) methods immediately after delivery including intrauterine devices (IUDs), implants, female sterilization, progesterone-only pills, LAM, and condoms [4].

LAM is a modern, short-term temporary contraceptive method based on natural infertility resulting from patterns of BF. Postpartum women who meet the following criteria can use LAM as an FP method: the first 6 months after birth, exclusive BF, and menstruation not resumed [4, 5]. There is a lack of awareness among Egyptian women in the postpartum period about the criteria for using LAM as a method of contraception. A survey done in Alexandria, Egypt, revealed that 35% of women with an unmet need for family planning mentioned lactation during the first year postpartum as a method to protect them from getting pregnant [6]. Moreover, the Egyptian Family Health Survey (EFHS) 2021 reported a higher rate, where 76.8% considered BF as an FP method [7].

In low- and middle-income countries such as Ghana, the utilization of modern PPFP increased from 29% in the immediate 1 month postpartum to 60% in the 12th month following birth [8]. In Egypt, PPFP use increases from 5% between 1 and 2 months postpartum to 62% at 12 months [9].

The most important factors influencing the use of PPFP in Egypt were socioeconomic status, education level of both partners, rural residency, misconceptions of the conditions for LAM, quality of service, inadequate health education about PPFP, and lack of training and supervision of community health workers [6, 10].

The postpartum period represents a significant opportunity for reaching women with effective FP methods. Women are more likely to engage with the health care system during ANC, delivery, postnatal care, and first-year infant immunizations. In Egypt, 100% of children receive at least one vaccination during their 1st year [11]. The most frequent visits or maybe the only visits to the health care facility are during the first year for routine immunization services. This is an important opportunity to discuss PPFP with postpartum women [12]. Moreover, 87% of women in Egypt deliver in a medical facility, and currently, 6% of women who deliver in a facility are using a modern method of family planning at 1 month postpartum (an indication of immediate PPFP provision) [11].

Integrating PPFP with other MCH services means offering contraceptive counseling and services as part of facility-based childbirth care before discharge from the health facility. Providing FP counseling as part of childbirth care raises awareness of the importance of birth spacing and postpartum contraceptive options [13].

Integrating PPFP counseling into pregnancy visits is cost-effective and efficient [14–16], 91% of women in Egypt receive ANC from a skilled provider, and PPFP counseling can be provided during ANC, helping couples develop a plan for contraceptive use after delivery [11]. The USAID Strengthening Egypt’s Family Planning Program (SEFPP) conducts a project in Egypt called post-partum/post-abortion contraception (PP/PAC) which promotes IUD insertion during cesarian section. This has become a routine service on a national scale as women are counseled, and IUDs are inserted immediately after delivery of the placenta [17].

According to the EFHS-2021, the Egyptian population was about 102 million, and the total fertility rate was 2.85 births/woman [7]. It is estimated that 9% of women of reproductive age in Egypt are postpartum in a given year, and 2% are postpartum and not using a modern method of contraception [11]. PPFP is a priority area and will help Egyptian women in achieving adequate birth intervals. There are few studies assessing the situation of PPFP especially in Upper Egypt. Even the Egyptian Demographic and Health Survey reports the utilization of contraception among married women of reproductive age and does not specifically address postpartum women.

Assiut Governorate is one of the largest governorates in Upper Egypt and has a high priority in FP programs. According to the EFHS-2021, the fertility rate in Upper Egypt was 3.30, contraceptive use was 59.1%, and the unmet need for FP was 16.8% [7]. A study in Assiut district reported higher contraceptive use (62.8%) and unmet needs (29%) [18]. The present study aims to identify utilization and factors associated with the use of PPFP in the Assiut Governorate and to assess the current status of integration of family planning counseling in the existing MCH services.

## Methods

### Study design

This is an analytical cross-sectional study conducted on 455 postpartum women in the reproductive age group from 15 to 49 years in the Assiut Governorate in Upper Egypt. Data collection was from 1 October 2020 to 28 February 2021.

### Sample size

The sample size was computed using the Stat-calc program of EPI-info version 7.2 using population survey or descriptive study calculation, with a confidence interval of 97.0%, acceptable margin of error of 5%, design effect of 1, and 62.0% PPFP utilization [9]. The minimum required sample size was calculated to be 444 and was increased to 455 participants.

### Sampling technique

Assiut Governorate is one of the largest governorates in Upper Egypt; it has a population of about 4.4 million, out of which 66% live under the poverty line. It consists of 11 districts (Abnub, Abu-Tig, El-Badari, El-Fath, El-Ghanayem, El-Qusiya, Assiut, Dairut, Manfalut, Sahel Selim, Sodfa) [19]. One district was chosen using simple random sampling from a covered sealed base and the chosen district was *Assiut district*. According to the data from the Egyptian Ministry of Health, Assiut district contains 16 urban and 28 rural PHC centers that offer routine immunization to children. Two urban (El-Walediah and Kolta) and 2 rural (Elwan and El Zawia) PHC centers were again chosen using simple random sampling from a covered sealed base consisting of all Assiut districts to conduct our study.

The numbers selected from each center were proportionate to the size of the population: 300 participants from the urban districts (150 from Kolta PHC and 150 from Elwalidia PHC), and 155 participants from the rural districts (75 from Elwan PHC and 80 from Elzawia PHC) were chosen.

### Selection criteria

The study was done on postpartum women between the ages 15 to 49 years who had given birth 12 months before the study and attended the selected PHC centers in Assiut district for child immunization at the age of 1 year (in order to report their contraceptive experiences during the first year after delivery). All women fulfilling the inclusion criteria were invited to participate in the study. Sexually inactive women as widows and divorcees and women who had undergone a hysterectomy were excluded from the study.

A modern contraceptive method is defined as a product or medical procedure that interferes with reproduction resulting from acts of sexual intercourse [20].

### The questionnaire

The study participants were interviewed using a semi-structured questionnaire translated into the Arabic language. The questionnaire was divided into four sections:

The first section detailed the participants’ demographic characteristics (e.g., age, residence, education, and working for cash) and socioeconomic level which was assessed using the Family Affluence Scale III (FAS III) [21].

The Family Affluence Scale III consists of six questions {own a car or another motorized vehicle (no = 0; yes, one = 1; yes, two = 2), presence of a separate bedroom (no = 0; yes = 1), number of computers (none = 0, one = 1; two = 2; more than two = 3), number of bathrooms (none = 0; one = 1; two = 2; more than two = 3), presence of a dishwasher (no = 0; yes = 1), number of times traveling for holiday/vacation last year (never = 0; once = 1; twice = 2; more than twice = 3)}. The responses to the items were summed to form a FAS index ranging from 0 to 13; the mean score of the FAS index was used to compare socioeconomic levels between the groups, with a higher score indicating a higher socioeconomic level.

The second section included women’s obstetric history: number of living children, number of male children, breastfeeding up to 12 months postpartum, and resumption of menstruation after the last delivery.

The third section included questions about PPFP knowledge, attitude, past and current use of contraception, the methods used, and the timing of the start of contraception use.

a The PPFP knowledge index

The PPFP knowledge index was created by asking seven questions to assess the women’s knowledge regarding the best time to start PPFP, criteria of exclusive breastfeeding, criteria of lactational amenorrhea method, time of the return of fertility during the postpartum period, return of fertility after stopping contraceptive utilization, whether PPFP can prevent unintended pregnancy, and knowledge of at least one modern contraceptive method that can be used during the first year after childbirth. Responses were given a score of 1 for a correct answer and a score of 0 for an incorrect/do not know answer. The scores of the seven questions were summed, ranging from 0 to 7, and a mean knowledge score was calculated with a higher score indicating better knowledge.

b Postpartum women’s attitudes toward PPFP [22]

The PPFP attitude index was created by asking ten questions on a 5-point Likert scale, with 1 point for strongly disagree and 5 for strongly agree, to assess the women’s attitude regarding the adoption of contraceptives in the future, encouraging her friends to adopt PPFP, discussing PPFP use with a partner, PPFP is good for mother and child health, exclusive breastfeeding used to prevent pregnancy, small family size makes family happy, PPFP use is good for the standard of living, using contraception can cause infertility, religion forbids contraceptive use, and using contraception is a shame. The score for the last three negative questions was reversed to 1 for strongly agree and 5 for strongly disagree; a higher score indicated a positive attitude.

The fourth section evaluated the current status of integrating PPFP counseling within antenatal, natal, and postnatal services.

The questionnaire was subjected to face validity, and forward–backward translation was conducted. Two forward translations of the Family Affluence Scale III into Arabic were performed: one by a professional translator and the other by a public health physician/professor at the faculty of commerce. Two back translations into English were done by two English-Arabic bilingual persons, who were blinded to the original English version. Additionally, the questionnaire was revised by two experts in reproductive health to check the clarity and relevance of the questions to the research objectives. The reliability of the used scales was assessed and the calculated Cronbach’s alpha for internal consistency was 0.72 in the FAS III index, 0.74 in the PPFP knowledge index, and 0.82 in the PPFP attitude index.

#### Pretest of the questionnaire

A pilot study was done on 10% of the sample size (40 postpartum women) not chosen from the selected sites of the data collection. It tested the feasibility of introducing the questionnaire to women at immunization time and whether the questionnaire required modifications or rewording. It was also used to estimate the time required for completing the questionnaire. Some questions were rephrased based on the pilot study. These pretested questionnaires were not included in the data analysis.

### Statistical analysis

Data analysis was performed using IBM-SPSS version 26.0. Qualitative data were expressed as frequencies and percentages while quantitative data were tested for normality by the Shapiro–Wilk test and expressed as the mean ± standard deviation or median and range according to the distribution of data. The outcome variable was the use of modern PPFP (yes = 1, and no = 0), and the explanatory variables were constructed after reviewing the available medical literature. Bivariate analyses using the chi-square/Fisher exact tests for comparing proportions and the independent sample *T*-test/Mann–Whitney *U* test for comparing the mean/median difference were used to test the associations between PPFP utilization and the explanatory variables. Multivariate analyses were performed to explore the most important predictors for PPFP utilization. All significant variables in the bivariate analysis were included in a backward LR logistic regression analysis. Adjusted odds ratios and 95% confidence intervals were reported for the constructed models. For all statistical tests, *p*-values of less than 0.05 were considered significant.

## Results

### Characteristics of the study participants

Table 1 shows the sociodemographic and some reproductive characteristics of the participants. The study included 455 postpartum women; their mean age was 26.87 ± 5.7 years, 68.1% were less than 30 years. More than half of them (56.7%) were from urban areas. About 67.0% had an educational level of secondary or above, 79.6% were not working for cash and 56.0% lived in nuclear families. The median socioeconomic level was 2.0 and ranged from 1 to 9. More than half (56.7%) of participants had < 3 children, and 78.5% had at least 1 male child. Most of them (87.5%) breastfed their children up to 12 months after childbirth. Menstruation returned in about 70% of them, with 60.7% of them experiencing the return of menstruation during the first 6 weeks postpartum.

**Table 1:** Characteristics of the study sample of postpartum women in Assiut, Upper Egypt, 2020–2021

Variable	*n* = 455	Percent
**Mother age in years**		
< 30	310	68.1
≥ 30 (30–43)	145	31.9
Mean ± SD (range)	26.87 ± 5.7 (16–43)	
**Residence**		
Urban	258	56.7
Rural	197	43.3
**Mother education**		
Below secondary	149	32.7
Secondary and above	306	67.3
**Women working for cash**		
Yes	93	20.4
No	362	79.6
**Family type**		
Nuclear	255	56.0
Extended	200	44.0
**Socioeconomic level (Family Affluence Scale III)**		
Median (range)	2.0 (1.0–9.0)	
**Living children**		
< 3 living children	258	56.7
≥ 3 living children	197	43.3
**Number of male children**		
Do not have	98	21.5
Have ≥ one male child	357	78.5
**Gender of the last child**		
Male	241	53.0
Female	214	47.0
**Breastfeeding up to 12 months postpartum of the last child**		
Yes	398	87.5
No	57	12.5
**Return of menstruation after the last delivery**		
Yes	318	69.9
No	137	30.1
**Time of menstruation return after the last delivery (*n* = 318)**		
During the first 6 weeks	193	60.7
After 3 months	56	17.6
After 6 months	45	14.2
After 9 months	16	5.0
At 12 months	8	2.5

Data were presented as frequency and (%) or mean ± standard deviation/median (range)

### Postpartum contraceptive practice and knowledge

About 37% of participants had used modern FP method before, and only 54.5% of participants currently used modern PPFP methods during the first year after childbirth, about 45.0% during the first 6 weeks postpartum, and about 24% of them waited till the return of menstruation to start contraceptive use. The most commonly used methods by postpartum women were IUDs (38.3%), oral contraceptives (23.8%), and injectable contraceptives (12.1%), and the least used methods were implants (8.1%) and condoms (2.0%) (Table 2).

**Table 2:** Postpartum contraceptive practice, knowledge, and attitude scores among the study sample of postpartum women in Assiut, Upper Egypt, 2020–2021

Variable	*n* = 455	Percent
**Ever used modern contraceptive methods before**		
Yes	168	36.9
No	287	63.1
**Current postpartum contraceptive use**		
Yes	248	54.5
No	207	45.5
**Time of starting contraceptive use (*n* = 248)**		
Immediately after birth at delivery time	11	4.4
After 6 weeks postpartum	111	44.8
When returned menstruation	59	23.8
After 3 months postpartum	25	10.1
After 6 months postpartum	27	10.9
After 9 months postpartum	8	3.2
At 1 year postpartum	7	2.8
**Method used by postpartum women (*n* = 248)**^**a**^		
Intrauterine device (IUD)	95	38.3
Oral contraceptive	59	23.8
Injectable contraceptive	30	12.1
Implants	20	8.1
Condom	5	2.0
**Husband participation in FP utilization (*n* = 248)**		
Yes	95	38.3
No	120	48.4
Did not discuss with him	33	13.3
**How husband participate? (*n* = 95)**^a^		
Buy method for her	50	52.6
Go with her to the doctor	46	48.4
Remind her	9	9.5
Use condom	5	5.3
**Reason for not using PPFP (*n* = 207)**^a^		
Husband opposition	67	32.4
Planning to get pregnant soon	48	23.2
Menstruation did not resume yet	45	21.7
Wants to get a male child	42	20.3
Methods-related health concern	40	19.3
**Knowledge score**		
Median (range)	3.0 (0.0–7.0)	
**Attitude score**		
Mean ± SD (range)	38.8 ± 5.56 (24–50)	

Data were presented as frequency and (%) or mean ± standard deviation/median (range)

^a^More than one answer was allowed

The women’s justifications for not using PPFP methods were mainly related to husband opposition (32.4%), planning to get pregnant soon (23.2%), menstruation did not resume (21.7%), wanting to get a male child (20.3%), and the method-related health concern (19.3%). Regarding the husband participation, only 38.3% of their husbands participated in PPFP use, where they participated by buying FP methods for their wives (52.6%), going with their wives to the doctor for the FP method (48.4%), and reminding her about the time of use of contraception (9.5%).

The total median PPFP knowledge score of the women was 3.0 and ranged from 0 to 7, and the mean attitude index score toward PPFP was 38.8 ± 5.56 with a range from 24 to 50.

### Integration of PPFP counseling with other MCH services

Although most of the participants (95.1%) attended four or more antenatal care visits (ANC), only 5.3% received FP counseling during those visits. Moreover, the vast majority of the women (91.9%) delivered their last child at the hospital, but only 1.3% received FP counseling during delivery. The majority of the women (87.9%) visited the PHC center for the first time postpartum for child vaccination, but only 3.5% received FP counseling during the postpartum period (Table 3).

**Table 3:** Status of integration of PPFP counseling with MCH services in Assiut, Upper Egypt, 2020–2021

Variable	*n* = 455	Percent
**Antenatal visits**		
Yes	451	99.1
No	4	0.9
**Number of antenatal visits (*n* = 451)**		
Less than 4	22	4.9
4 and more	429	95.1
**Received FP counseling during antenatal visits (*n* = 451)**		
Yes	24	5.3
No	427	94.7
**Place of antenatal visits (*n* = 451)**		
Primary health care centers	171	37.9
Public hospital/university hospital	22	4.9
Private clinic	285	63.2
**Place of delivery for the last child**		
Hospital (public/university/private)	418	91.9
Home with a trained nurse	37	8.1
**Received FP** c**ounseling during delivery**		
Yes	6	1.3
No	449	98.7
**First visit to primary health care center**		
Vaccination	400	87.9
Child follow-up	36	7.9
Mother follow-up	19	4.2
**Received FP counseling during the postpartum period**		
Yes	16	3.5
No	439	96.5

### Predictors of using PPFP methods

Bivariate analysis revealed that the use of modern PPFP methods among the studied women was significantly associated with being older, residing in urban areas, having secondary education or above, working for cash, living in a nuclear family, and having a high score for the socioeconomic level. It was also associated with having three or more children and at least one male child. Moreover, it was associated with the resumption of menstruation after the last delivery, prior usage of modern FP methods, and having better reproductive health knowledge and attitude toward PPFP.

The use of PPFP methods was also associated with receiving information from health care providers and social media. Moreover, it was associated with attending four or more ANC visits, receiving FP counseling during ANC visits, delivering the last child at the hospital, and receiving FP counseling at postpartum visits to health care facilities (Table 4).

**Table 4:** Factors affecting the use of PPFP among study participants in Assiut, Upper Egypt

Variable	Users (*n* = 248), no. (%)	Non-users (*n* = 207), no. (%)	*p*-value
**Age of the mother (years)**			
Mean ± SD	28.8 ± 5.3	24.7 ± 5.5	< 0.001*
**Residence**			
Urban	193 (74.8)	65 (25.2)	< 0.001*
Rural	55 (27.9)	142 (72.1)	
**Education level**			
Below secondary education	35 (23.5)	114 (76.5)	< 0.001*
Secondary and above	213 (69.6)	93 (30.4)	
**Working for cash**			
Yes	69 (74.2)	24 (25.8)	< 0.001*
No	179 (49.4)	183 (50.6)	
**Type of family**			
Nuclear family	178 (69.8)	77 (30.2)	< 0.001*
Extended family	70 (35.0)	130 (65.0)	
**Socioeconomic level: median (range)**	2.5 (1.0–9.0)	1.0 (1.0–7.0)	< 0.001*
**Living children**			
< 3 living children	129 (50.0)	129 (50.0)	0.027*
≥ 3 living children	119 (60.4)	78 (39.6)	
**Having at least one male child**			
Do not have	44 (44.9)	54 (55.1)	0.031*
Have male children	204 (57.1)	153 (42.9)	
**Breastfeeding up to 12 months postpartum**			
Yes	219 (55.0)	179 (45.0)	0.556
No	29 (50.9)	28 (49.1)	
**Resumption of menstruation after the last delivery**			
Yes	195 (61.3)	123 (38.7)	< 0.001*
No	53 (38.7)	84 (61.3)	
**Ever used contraceptive methods before**			
Yes	145 (86.3)	23 (13.7)	< 0.001*
No	103 (35.9)	184 (64.1)	
**Knowledge score: median (range)**	4.0 (1.0–7.0)	3.0 (0.0–6.0)	< 0.001*
**Attitude score: mean ± SD**	42.2 ± 4.0	34.7 ± 4.2	< 0.001*
**Source of information**			
Family and friends	149 (60.0)	185 (89.4)	< 0.001*
Health care provider	135 (54.4)	9 (4.3)	< 0.001*
Social media	77 (31.0)	22 (10.6)	< 0.001*
**Antenatal care**			
Yes	247 (54.8)	204 (45.2)	0.334
No	1 (25.0)	3 (75.0)	
**Number of antenatal visits (*n* = 451)**			
Less than 4	0 (0.0)	22 (100.0)	< 0.001*
4 and more	247 (57.6)	182 (42.4)	
**Received FP counseling during antenatal visits (*n* = 451)**			
Yes	22 (91.7)	2 (8.3)	< 0.001*
No	225 (52.7)	202 (47.3)	
**Place of the last delivery**			
General or private hospital	235 (56.2)	183 (43.8)	0.014*
Home	13 (35.1)	24 (64.9)	
**Received FP** c**ounseling during delivery**			
Yes	5 (83.3)	1 (16.7)	0.227
No	243 (54.1)	206 (45.9)	
**Received FP counseling at postpartum visit to the health care facility**			
Yes	13 (81.2)	3 (18.8)	0.029*
No	235 (53.5)	204 (46.5)	

The chi-square or Fisher exact test was used to compare the proportions and the independent samples *T* test/Mann–Whitney *U* test was used to compare the means/median differences between users and non-users

^*^Significant variable (*p*-value < 0.05)

Table 5 shows the results of the backward LR regression analysis. When health care providers are the source of PPFP information, the use of PPFP increased 11 times compared to those who did not receive that information (*p* < 0.001). Moreover, better attitude towards PPFP (AOR = 10.54, *p* < 0.001), prior use of modern FP methods (AOR = 6.98, *p* < 0.001), resumption of menstruation (AOR = 4.11, *p* < 0.001), older age (AOR = 2.15, *p* < 0.05), and better PPFP knowledge (AOR = 1.72, *p* < 0.01) were significant predictors for PPFP utilization.

**Table 5:** Predictors of PPFP utilization among study participants in Assiut, Upper Egypt

Predictor	*B*	SE	AOR, 95% CI (lower–upper)	*p*-value
Receiving information about PPFP from health care provider	2.44	0.46	11.46 (4.65–28.25)	< 0.001*
Attitude score	2.36	0.33	10.54 (5.54–20.03)	< 0.001*
Ever used the modern FP method before	1.94	0.36	6.98 (3.44–14.18)	< 0.001*
Resumption of menstruation	1.41	0.36	4.11 (2.02–8.38)	< 0.001*
Women age	0.77	0.37	2.15 (1.04–4.46)	0.039*
Knowledge score	0.54	0.16	1.72 (1.26–2.36)	0.001*

The table is based on backward LR logistic regression to identify predictors of PPFP utilization

*CI* confidence interval, *AOR* adjusted odds ratio, *SE* standard error

^*^Significant predictor (*p*-value < 0.05)

## Discussion

Assiut is one of the nine Upper Egypt governorates in which the Strengthening Egypt’s Family Planning Program was implemented (2018–2022) to decelerate Egypt’s rapid population growth. The present study assessed the use of PPFP during 2020–2021 among a representative sample of postpartum women in Assiut governorate. Our results showed a lower rate of use (54.4%) compared to other studies in Egypt, for example, the current FP use revealed by the EFHS 2021 [7] was 66.4%, and modern PPFP use at 6 months after delivery in Egypt 2021 by Track 20 was 74% [11].

Moreover, PPFP use was 51.8% among women attending family medicine units in 6th October City, Egypt [23], and 61.8% among women in Minia District, Egypt [24]. However, in Alexandria, Egypt, it was 83.7% [6]. The variation might be due to variations in the sociodemographic and reproductive characteristics of the study population as Alexandria is an urban community in comparison with the other mentioned sites which contain both urban and rural areas.

Egyptian women prefer the IUD as it is the most effective reversible method, does not require self-timing to be remembered, and is easily accessible in PHC units with trained doctors. The use of IUDs was followed by the use of pills as it is convenient with no need for doctor intervention and can be used for short term [7, 23]. This is consistent with our results as IUDs followed by oral and injectable contraceptives were the most commonly used methods, while implants and condoms were the least used methods.

Less than half of the participants in our study used PPFP at 6 weeks postpartum while more than one-fifth waited for menstruation to return, and the least proportion started contraception immediately after delivery. This result is consistent with the results reported in Addis Ababa, Ethiopia, and Kailali District, Nepal [25, 26].

The most reported reasons for not using PPFP in our study were husband opposition, planning to get pregnant soon, menstruation did not resume, and wanting to get a male child. This finding indicated the importance of focusing on male involvement in family planning efforts because husbands play a role in deciding future contraceptive methods used by their wives. Aside from the husband’s opposition which was a key reason, our results are consistent with a study in Alexandria, Egypt, and a study in Minia District, Egypt, that reported that amenorrhea, breastfeeding, fear of side effects, health concerns, and surrounding social pressure were the most common reasons for not using PPFP [6, 24].

PPFP utilization significantly increases with older maternal age. This is consistent with the study done in Awish El-Hagar village, Mansoura district, Dakahlia Governorate, Egypt, and supported by the results of the EFHS-2021 [7, 27]. This may be explained by the fact that too many pregnancies at old age expose women to pregnancy-related complications.

PPFP utilization significantly increases among women living in urban areas. This is consistent with the study in Alexandria, Egypt [6]. The difference in utilization between urban and rural may be due to the difference in educational level, surrounding social pressure, and lack of women empowerment in rural and semi-rural communities.

Women with higher educational levels, working for cash, and with higher socioeconomic levels have higher PPFP utilization. This is supported by other studies in Egypt [6, 24, 27], along with the results of a systematic review on postpartum contraceptive use among women in low- and middle-income countries [28], and by the results in Ghana [8]. Educated women mostly working for cash and with higher socioeconomic levels are more likely to visit a health facility and have a better understanding of the available methods of family planning during the postpartum period.

Moreover, in our study, women who had at least one male child were more likely to use FP methods. This may be due to gender preference as most families in Arab countries prefer to have a male child. This is consistent with the study done in Mansoura, Egypt [27]. Women who had previously used modern FP methods were six times more likely to use PPFP. This is consistent with the studies conducted in Kailali District, Nepal, and Addis Ababa, Ethiopia [29, 30].

Women with postpartum amenorrhea were less likely to use contraceptive methods, while the resumption of menstruation was significantly associated with higher PPFP use. This is consistent with the results of a systemic review on postpartum contraceptive use among women in low- and middle-income countries [28]. Women believe that the return of menses is a sign of the return of fertility and does not realize that ovulation may precede the appearance of menses.

Health care providers were the main source of information, which is consistent with the results in Minia District, Egypt, and the EFHS-2021 [7, 24]. In our study, when health care providers were the source of PPFP information, the use of PPFP increased 11 times compared to those who did not receive such information. This indicates that health care providers play an essential role in influencing FP uptake. This is consistent with the studies conducted in the Somali Region, Ethiopia, and Addis Ababa, Ethiopia [22, 25].

Moreover, in our study, increasing PPFP knowledge was a significant predictor for PPFP utilization. In Egypt, 1.8 months is the median duration of exclusive breastfeeding. Increasing women’s knowledge through education and counseling on the effective use of LAM during ANC, post-natal care, and infant health-related services can increase the use of this method [11]. According to the EFHS-2021, 14% of currently married women in Egypt have an unmet need for FP [7]. Cultural norms about the contraceptive effects of lactation and the inaccurate knowledge of women about the conditions for appropriate use of the LAM as a contraceptive method were the most reported reasons for the unmet need for PPFP in a study done in Alexandria, Egypt [10].

Our study revealed that a positive attitude toward the utilization of PPFP was significantly associated with PPFP utilization. The same results were reported in Addis Ababa and Kebribeyah Town in Ethiopia [22, 25].

Although more than 95% of participants attended four or more antenatal visits, only 5.3% of them received PPFP counseling during ANC visits. Women attending four or more ANC visits were more likely to use PPFP. This is consistent with the results of a systematic review on postpartum contraceptive use among women in low- and middle-income countries [28]. Women who attend ANC are more likely to get information about contraceptive use.

While most women delivered their last child in a health facility, only 1.3% received PPFP counseling during delivery. This implies that there is a missed opportunity as the delivery period is an important time whereby most women are supposed to get counseling on PPFP.

PPFP utilization significantly increases with delivery in health facilities. In Egypt, 6% of women who deliver in a facility are using a modern method at 1 month postpartum which is an indication of immediate PPFP provision [11]. Expanding post-natal checkups and integrating PPFP counseling with delivery and follow-up care can improve PPFP uptakes.

Only 3.5% of participants received FP counseling during the postpartum period, and they were more likely to use PPFP methods, which is consistent with the results from Ghana [8]. This indicates that counseling about PPFP during postnatal care can encourage women to utilize the FP service during the postpartum period. The lack of information can place women at risk of unwanted pregnancy soon after a previous birth [31].

### Limitations

The sensitive nature of data related to contraceptive use could be a limitation as respondents may be resistant to providing certain information, which they may consider to be intimate. This limitation was addressed by assuring confidentiality. Factors related to the health system and the service providers were not included in the current study. The cross-sectional design used has some degree of recall bias; however, this was addressed by recruiting women who had given birth 12 months prior to the study.

## Conclusions

Postpartum contraception use was found to be associated with receiving PPFP counseling by health care providers and women’s knowledge and attitude regarding PPFP. However, FP counseling was not found to be integrated with other MCH services. Updating the components of MCH services to include PPFP counseling during ANC, at delivery, and during the postpartum period should be prioritized by program planners and policymakers.

## Data Availability

The datasets generated and analyzed during the current study are not publicly available due to privacy considerations of the participants but are available from the corresponding author upon reasonable request.

## Abbreviations

PPFP: Postpartum family planning
ANC: Antenatal care
MCH: Maternal and child health
PHC: Primary health care centers
EFHS: Egyptian Family Health Survey
